# LASER: A Maximum Likelihood Toolkit for Detecting Temporal Shifts in Diversification Rates From Molecular Phylogenies

**Published:** 2007-02-14

**Authors:** Daniel L Rabosky

**Affiliations:** Department of Ecology and Evolutionary Biology, Cornell University, Ithaca, NY 14853-2701, U.S.A

**Keywords:** Maximum likelihood, diversification, birth-death process, phylogeny

## Abstract

Rates of species origination and extinction can vary over time during evolutionary radiations, and it is possible to reconstruct the history of diversification using molecular phylogenies of extant taxa only. Maximum likelihood methods provide a useful framework for inferring temporal variation in diversification rates. LASER is a package for the R programming environment that implements maximum likelihood methods based on the birth-death process to test whether diversification rates have changed over time. LASER contrasts the likelihood of phylogenetic data under models where diversification rates have changed over time to alternative models where rates have remained constant over time. Major strengths of the package include the ability to detect temporal increases in diversification rates and the inference of diversification parameters under multiple rate-variable models of diversification. The program and associated documentation are freely available from the R package archive at http://cran.r-project.org.

## Introduction

Recent years have seen an explosive proliferation of DNA sequence data for molecular phylogenetic analyses and a commensurate increase in the use of these data to draw inferences about macroevolutionary processes. A particularly active area of research involves the use of molecular phylogenies to study variation in rates of species origination and extinction, both among lineages (Slowinski and Guyer, 1989; Mooers and Heard, 1997) and over time (Nee et al. 1994; Paradis, 1997).

Likelihood Analysis of Speciation and Extinction Rates (LASER) is a package for the R programming environment that facilitates model-based analyses of diversification rates. LASER is the first software package to implement tests for temporal variation in diversification rates using likelihood methods based on the birth-death process (Nee et al. 1994). LASER is licensed under the GNU General Public License and complements the existing R libraries ‘ape’ (Paradis et al. 2004) and ‘apTreeshape’ (Bortolussi et al. 2006), which provide functions for phylogenetic tree manipulation and the analysis of among-lineage heterogeneity in diversification rates.

LASER was written to address several limitations of existing software for analyzing the tempo of diversification. Approaches such as the gamma statistic (Pybus and Harvey, 2000) and survival analysis (Paradis, 1997), which are implemented in the R library ‘ape’ (Paradis et al. 2004), test for departures from the pure-birth model of cladogenesis, and can only be used to infer temporal decreases in diversification rates (Nee, 2001; Rabosky, 2006). These methods are thus unable to address many questions of interest to evolutionary biologists, such as whether temperate faunas experienced elevated speciation rates during the Pleistocene (Weir and Schluter, 2004). Furthermore, existing methods suffer reduced power to detect temporal decreases in diversification rates when clades have diversified under high background extinction rates (Rabosky 2006).

LASER fits a candidate set of rate-variable diversification models to phylogenetic data and contrasts the likelihood of the data under these models to alternatives where speciation and extinction rates have remained constant over time. The null hypothesis that diversification rates have not changed over time is tested using the statistical approach described in Rabosky (2006). The test statistic for constancy of diversification rates is computed as

$$\Delta {\text{AIC}}_{\text{RC}}={\text{AIC}}_{\text{RC}}-{\text{AIC}}_{\text{RV}}$$

where AIC_RC_ is the Akaike Information Criterion (AIC) score for the best-fit rate-constant model of diversification, and AIC_RV_ is the AIC score for the best-fit rate-variable model under consideration. Thus, a positive ΔAIC_RC_ value suggests that the data are best approximated by a rate-variable model of diversification. Although several previous studies have used the AIC to distinguish among rate-constant and rate-variable models of diversification (Barraclough and Vogler, 2002; Turgeon et al. 2005), Rabosky (2006) found that this approach results in high Type I error rates unless critical values of the ΔAIC_RC_ distribution are explicitly addressed through simulation.

The LASER package provides a comprehensive toolkit for computing ΔAIC_RC_ for test phylogenies and for comparing the observed ΔAIC_RC_ statistic to its distribution under the null hypothesis. This is the first available approach that can detect temporal increases in diversification rates, and extensive simulation has shown that the method has greater power than other methods to detect temporal declines in diversification rates when clades have diversified under elevated background extinction rates (Rabosky, 2006).

Additional strengths of the model-fitting approach implemented in the LASER include the ability to test hypotheses of rate variation while estimating relevant diversification parameters. Furthermore, the package can be used to test *a priori* hypotheses of temporal rate variation. The R programming environment used by LASER provides great flexibility, and likelihood functions in the package can easily be tailored to a variety of statistical applications. For example, one can generate posterior distributions of diversification parameters using the posterior distribution of phylogenetic tree topologies and branch lengths sampled using Markov chain Monte Carlo (MCMC) methods (e.g. Huelsenbeck and Ronquist, 2001).

## Usage

LASER operates on sets of branching times derived from ultrametric phylogenetic trees, and provides functions for obtaining branching times from several input formats, including the widely used ‘Newick’ (parenthetic) tree format. Likelihoods and parameter estimates can be obtained for a range of rate-variable diversification models, including logistic and exponential density-depedent models and multi-rate birth-death models. Additional functions permit batch processing of multiple phylogenies to obtain the null distribution of ΔAIC_RC_ or posterior distributions of diversification parameters.

The function fitdAICrc computes the ΔAIC_RC_ test statistic for a test phylogeny using arguments that specify the candidate set of rate-variable models to be considered. The null distribution of the test statistic is obtained by either simulating branching times with the function yuleSim or by importing simulated trees using the function getBtimes.batch. The latter function is particularly useful for the analysis of phylogenies with incomplete taxon sampling, because incomplete sampling can result in a spurious decline in diversification rates over time (Pybus and Harvey, 2000). To address this problem in LASER, one can simply generate rate-constant phylogenies with incomplete sampling using PhyloGen (Rambaut, 2002) or other software and import the trees into LASER to tabulate the null distribution of the ΔAIC_RC_ test statistic.

A call to the function fitdAICrc.batch will then generate the null distribution of the test statistic and return the probability of the observed ΔAIC_RC_ index under the null hypothesis. Functions are available to call any rate-variable and rate-constant diversification models individually, and additional functions permit exploration of diversification patterns for any user-defined temporal interval.

## Diversification Models

Rate-constant diversification models implemented in LASER include the pure birth model, with a constant speciation rate λ > 0, and the birth-death model, with λ > 0 and extinction rate μ ≥ 0. Seven rate-variable diversification models are provided, including density-dependent and multi-rate variants of the pure birth and birth-death models. The package includes both logistic and exponential density-dependent speciation models. Under the logistic density-dependent model of cladogenesis, the speciation rate λ at time *t* is modeled as

$$\lambda (t)=\lambda_{0}\left(1-\frac{N_{t}}{K}\right)$$

where λ_0_ is the initial speciation rate, *N_t_* is the number of lineages at time *t* in a reconstructed phylogeny, and *K* is analogous to the carrying capacity parameter of population ecology. Speciation rates are modeled under an exponential density-dependent process as

$$\lambda (t)=\lambda_{0}N_{t}^{-x}$$

where *x* controls the magnitude of the rate change with respect to the number of lineages at any point in time in the reconstructed phylogenetic tree.

Multi-rate variants of the pure birth and birth-death model assume the existence of one or more breakpoints in time, such that a clade has diversified under one set of diversification parameters before the breakpoint and another set of parameters after the breakpoint. For example, LASER includes a two-rate pure birth model with three parameters: the initial speciation rate, the final speciation rate, and the time of the rate shift.

## Example: Holarctic Damselfly Radiation

Turgeon et al. (2005), tested whether Holarctic damselflies in the genus *Enallagma* showed evidence for increased diversification rates during the Quaternary. They found evidence for a recent increase in speciation rates, suggesting a role for Pleistocene glacial cycles in damselfly diversification. Their conclusions were based on a model-fitting approach similar to that described above. However, as noted in Rabosky (2006), this method can result in high Type I error rates. To explicitly address this problem, I used LASER to compute ΔAIC_RC_ for the *Enallagma* phylogeny from Turgeon et al. (2005) and to tabulate the null distribution of the statistic as follows:

data.bt <- getBtimes(file = ‘enallagma.tre’) 

summary <- fitdAICrc(data. bt) 

The first line creates a vector data.bt of the branching times for *Enallagma* by reading the parenthetic tree stored in enallagma.tre. The function fitdAICrc generates an object, summary, that contains the results of fitting all rate-variable and rate-constant models to the data. The observed ΔAIC_RC_ statistic for *Enallagma* is 13.0372, and the best fit model is a three-parameter rate-variable model specifying a 12.5-fold increase in the speciation rate over time. The significance of the observed ΔAIC_RC_ statistic was assessed with the following commands:

null.bt <- yuleSim(37,5000) 

fitdAICrc.batch (null.bt, stat = 13.0372) 

**Figure 1:**
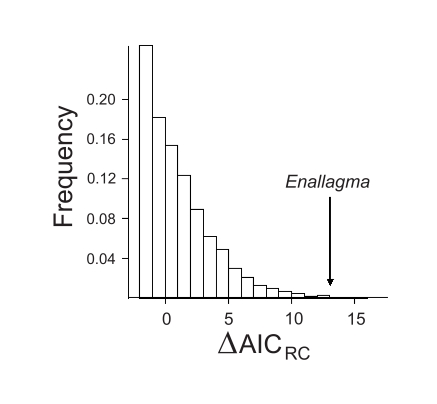
Distribution of the ΔAIC_RC_ test statistic for 5000 rate-constant phylogenies of the same size as the *Enallagma* phylogeny. The calculated ΔAIC_RC_ for *Enallagma* was 13.0372 and indicates a highly significant temporal increase in the net diversification rate over time (p = 0.0016).

The first line simulates 5000 phylogenies with the same number of tips as the *Enallagma* tree (37) under the null hypothesis of rate-constancy and stores them in object null.bt. The set of simulated phylogenies is analyzed using fitdAICrc. batch, which approximates the probability of the observed ΔAIC_RC_ statistic under the null hypothesis. In this case, the observed ΔAIC_RC_ statistic indicates a highly significant departure from the null hypothesis of rate-constancy (p = 0.0016; Fig. 1), supporting the conclusions of Turgeon et al. (2005).

## Conclusion

LASER fits multiple rate-variable and rate-constant models of diversification to reconstructed phylogenies using maximum likelihood. Its main strength includes the use of Monte Carlo simulation to control for elevated Type I error rates associated with likelihood-based analyses of diversification. LASER is the first available package that can detect temporal increases in diversification rates, and has considerable power to detect temporal declines in diversification rates when clades have diversified under high background extinction rates. As a freely available package for the R programming environment, it is flexible and platform-independent, and can easily be tailored to a variety of user-specific applications.

## References

[b1-ebo-02-273] Barraclough TG, Vogler AP (2002). Recent diversification rates in North American tiger beetles estimated from a dated mtDNA phylogenetic tree.. Mol. Biol. Evol.

[b2-ebo-02-273] Bortolussi N, Durand E, Blum MGB, Francois O (2006). APTreeshape: Statistical analysis of phylogenetic tree shape. Bioinformatics.

[b3-ebo-02-273] Huelsenbeck JP, Ronquist F (2001). MrBayes: Bayesian inference of phylogeny. Bioinformatics.

[b4-ebo-02-273] Mooers A, Heard SB (1997). Evolutionary process from phylogenetic tree shape.. Q. Rev. Biol.

[b5-ebo-02-273] Nee S (2001). Inferring speciation rates from phylogenies. Evolution.

[b6-ebo-02-273] Nee S, May RM, Harvey PH (1994). The reconstructed evolutionary process.. Phil. Trans. R. Soc. Lond. B Biol. Sci.

[b7-ebo-02-273] Paradis E (1997). Assessing temporal variations in diversification rates from phylogenies: estimation and hypothesis testing.. Proc. R. Soc. Lond. B Biol. Sci.

[b8-ebo-02-273] Paradis E, Claude J, Strimmer K (2004). APE: An R Package for Analyses of Phylogenetics and Evolution. Bioinformatics.

[b9-ebo-02-273] Pybus OG, Harvey PH (2000). Testing macro-evolutionary models using incomplete molecular phylogenies.. Proc. R. Soc. Lond. B Biol. Sci.

[b10-ebo-02-273] Rabosky D (2006). Likelihood methods for inferring temporal shifts in diversification rates. Evolution.

[b11-ebo-02-273] Rambaut AP (2002).

[b12-ebo-02-273] Slowinski JG, Guyer CG (1989). Testing the stochasticity of patterns of organismal diversity: an improved null model. American Naturalist.

[b13-ebo-02-273] Turgeon J, Stoks R, Thum RA, Brown JM, McPeek MA (2005). Simultaneous quaternary radiations of three damselfly clades across the Holarctic. American Naturalist.

[b14-ebo-02-273] Weir JT, Schluter D (2004). Ice sheets promote speciation in boreal birds.. Proc. R. Soc. Lond. B Biol. Sci.

